# Novel insight into PINK1/parkin-associated autophagy implicated in Parkinson disease

**DOI:** 10.1515/tnsci-2025-0386

**Published:** 2026-04-20

**Authors:** Judith Stegmüller

**Affiliations:** Department of Neurology, RWTH University Hospital, Pauwelsstrasse 30 52074, Aachen, Germany

**Keywords:** Parkinson disease, PINK1, parkin, mitophagy, autophagy.

## Abstract

Parkinson disease (PD) and its variants pose a dramatic burden on patients, families and society. Deciphering the mechanistic underpinnings of PD are critical goals of researchers to develop new therapeutic approaches. Among the pathways affected, autophagy draws increasing attention owing to its relationship to several genes implicated in PD and parkinsonism. This review summarizes novel insight into autophagic and in particular mitophagic processes regulated by parkin and PINK1, and how their deregulation may contribute to or cause the disease.

## Parkinson disease

Parkinson disease (PD) is a progressive neurodegenerative disorder. PD patients display motor symptoms including resting tremor, bradykinesia and rigidity, and they also suffer from a wide array of non-motor symptoms. The latter include abnormal sensation such as hyposmia and pain, behavioral changes such as depression and apathy, autonomic dysfunction such as cardiovascular issues, sleep disturbances, fatigue and cognitive deficits [1]. At the histological level, PD is characterized by the loss of dopaminergic neurons, which trigger motor symptoms, and by Lewy bodies [2].

A recent meta-analysis impressively demonstrated why PD research is more relevant than ever. Thoroughly covering over 40 years of PD patient data, temporal monitoring revealed almost a triplication of the cases between 2010 and 2023, a higher prevalence across WHO (world health organization) regions with higher living standard, health and education, and a staggering increase in cases among individuals older than 60 years [3].

The discovery of 18 *PARK* loci associated with familial PD over the past three decades provides valuable insight into the molecular underpinnings of PD [4]. The identification of mutations in *PARK2* and *PARK6* encoding for the E3 ubiquitin ligase parkin and the kinase PINK1, respectively, revealed players in autosomal recessive parkinsonism variants [5], 6], which are characterized by early-onset and slow progression. Research launched over a decade ago presented a critical role for parkin and PINK1 in the regulation of mitophagy, a specialized variant of macroautophagy (referred to as autophagy hereafter) [7], 8].

## A brief introduction to autophagy as well as parkin- and PINK1-regulated mitophagy

Autophagy is a highly conserved, cellular “self-eating” system, that is controlled by many autophagy-related genes (*ATG)*, and that mediates the degradation of cytoplasmic components and organelles (for in-depth reviews see: [9], [10], [11]). Briefly, the initiation step of autophagy requires the ULK1/2 initiation complex which triggers phosphorylation events required for the nucleation of the isolation membrane (also referred to as phagophore). The cargo is captured by the isolation membrane, decorated with lipidated microtubule-associated protein 1 light chain 3 molecules (short: LC3). The closure of the double membrane and thus the engulfment of the cargo, that includes bacteria, proteins, protein aggregates, ribosomes, endoplasmic reticulum or mitochondria, completes the formation of the autophagosomes. This is then followed by the fusion with lysosomes and the maturation into autolysosomes, which launches the degradation process. Mitophagy describes mitochondrial autophagy and requires a specific set of receptor proteins in a ubiquitin-independent manner or sophisticated ubiquitination events in need of E3 ubiquitin ligases such as parkin.

PINK1 (PTEN-induced kinase 1) and parkin are two key proteins involved in the process of mitophagy. PINK1 is a mitochondrial serine/threonine kinase that is targeted to healthy mitochondria, but rapidly degraded. However, when mitochondria become depolarized or damaged, PINK1 accumulates on the outer mitochondrial membrane and becomes an active kinase via autophosphorylation. This leads to the recruitment of parkin to mitochondria and the phosphorylation of ubiquitin. Parkin ubiquitinates various outer mitochondrial membrane (OMM) proteins, which results in the extraction of ubiquitinated mitochondrial areas, and importantly, in the recognition and clearance of impaired mitochondria.

This review is hence dedicated to new insights into the specific functions of the E3 ligase parkin and the kinase PINK1, and into deubiquitinase-mediated regulation of parkin activity. It further highlights new aspects of upstream regulation of PINK1/parkin autophagy, in addition to parkin’s and PINK1’s interaction with other *PARK*-encoded proteins or the ubiquitin-proteasome system. Lastly, interesting novel therapeutic approaches targeting parkin and PINK1 are featured.

## What’s new with parkin?

**Figure 1:**
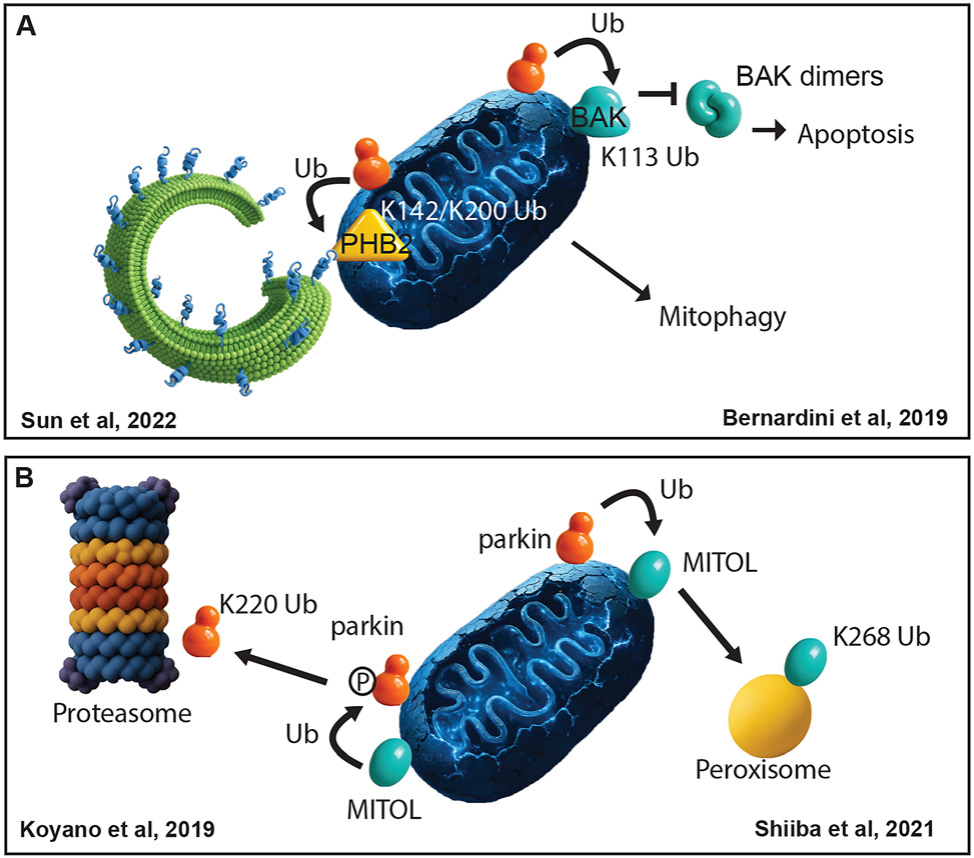
A. Sun and colleagues described the ubiquitination of the mitochondrial chaperone prohibitin 2 by parkin, which leads to a higher affinity for the isolation membrane. Bernardini and colleagues described that mitochondrial damage leads to ubiquitinated BAK which prevents its dimerization and promotes mitophagy. B. Koyano and colleagues identified parkin as a target of the E3 ubiquitin ligase MITOL, which leads to the proteasomal degradation of parkin. Shiiba and colleagues found that parkin ubiquitinates MITOL, which is sorted away from damaged mitochondria to peroxisomes.

The current paradigm of parkin activity is that ubiquitination of components of the OMM is the priming event for mitophagy. The identification of these proteins is key to understanding the ramifications of PINK1/parkin-regulated mitophagy. Previously, prohibitin 2 (PHB2), a chaperone localized at the inner mitochondrial membrane (IMM), was identified as a mitophagy receptor that requires the rupture of the outer mitochondrial membrane for its interaction with LC3 at the isolation membrane [12]. A recent study followed up and revealed that PHB2 is a novel target of parkin and undergoes K11- and K33-linked ubiquitination [13], (Figure 1A). This results in higher affinity of PHB2 for LC3 at the autophagosome. Upon induction of mitophagy, parkin also ubiquitinates BAK (BCL-2 antagonist/killer-1) in a nonproteolytic manner and leads to reduced levels of BAX (BCL-2-associated X protein) [14], (Figure 1A). BAK together with BAX are proapoptotic BH3-only proteins, which homodimerize. Homodimers then multimerize and perforate the outer mitochondrial membrane, leading to necessary rupturing events during apoptosis [15]. Parkin has earlier been demonstrated to ubiquitinate BAK in the context of apoptosis [16]. Ubiquitination of BAK inhibits its activity and oligomerization, thus preventing cell death-triggering formation of BAK oligomers [14]. Additionally, Bernardini et al. found that BAK oligomers stimulate the activation of PINK1/parkin-dependent mitophagy as a consequence of BAK’s permeabilization effect on the OMM during apoptosis. Collectively, BAK is a non-canonical mitophagy substrate, but cellular stress can drive its ubiquitination by parkin that prevents oligomerization, thereby limiting apoptosis and allowing for mitochondrial clearance in the cell.

Aside from parkin, MITOL is another E3 ubiquitin ligase active on mitochondria. MITOL was recently reported to specifically ubiquitinate phosphorylated parkin at K220 upon mitophagy [17], (Figure 1B). As a result, parkin is targeted for proteasomal degradation. By degrading parkin, MITOL indirectly protects cells from apoptosis by sparing the peptidyl-prolyl cis-trans isomerase FKBP38 from parkin-mediated degradation. Owing to FKBP38’s association with the pro-survival BH3-only protein BCL-2, FKBP38 exerts its anti-apoptotic activity in the cell. Furthermore, MITOL plays a role in boosting the low target specificity of parkin towards its substrates on mitochondria [18], 19]. The latter was shown by the ubiquitination of mitochondria-targeted, artificial substrates of parkin and the lag in parkin activity on mitochondria in the absence of MITOL. Another fascinating aspect of parkin activity, reported by the same lab, leads to the redistribution of proteins away from mitochondria destined for mitophagy. In this context, the E3 ligases swap roles and MITOL becomes ubiquitinated by parkin at K268. This serves as a sorting signal to trigger MITOL’s relocation to peroxisomes that is supported by peroxins (peroxisomal biogenesis factor 3 and 16) [18], 19], (Figure 1B). This novel process can be viewed as a way for specialized proteins to get away from damaged mitochondria and escape unwanted degradation.

Further substrates of parkin include MIRO1/2 proteins, which are atypical Rho GTPases with Ca^2+^-binding domains (aka: EF hand). They are subunits of an adaptor complex that mediates the transport of mitochondria along microtubules in long axons (in-depth reviews: [20]). MIRO proteins were formerly reported to directly interact with PINK1 [21]. Birsa and colleagues also showed that ubiquitination of MIRO1 is triggered by mitochondrial damage in a PINK1- and parkin-dependent fashion. In the following study, the authors demonstrated that a subset of parkin molecules interacts with MIRO1 in the absence of mitochondrial dysfunction. Upon mitochondrial damage and PINK1 accumulation however, the aforementioned pool of parkin proteins undergoes activation leading to the ubiquitination of MIRO1 [22]. Remarkably, loss of MIRO1 inhibits the translocation of parkin to mitochondria and suppresses mitophagy, indicating a role for MIRO1 as a parkin docking station. The EF hands of MIRO1, which confer Ca^2+^ sensitivity, play a critical role in its ubiquitination, in parkin recruitment to damaged mitochondria, and consequently, in glutamate-triggered mitophagy. To a similar conclusion led the study by López-Doménech and colleagues, who found that lack of MIRO1/2 leads to delayed translocation of parkin to dysfunctional mitochondria and their insufficient removal in neurons [23]. These findings were bolstered in mice with MIRO1 deletion in hippocampus and cortex, that show engorged and hyperfused mitochondria, enrichment of mitofusin and the activation of a set of kinases characteristic for integrated stress response, a process that slows down protein synthesis.

An astounding revelation brought about a study by Moskal et al. as they identified the well-established inhibitor of ROCK (Rho kinase) as a stimulator of parkin-mediated autophagy [24]. ROCK1 and its homologue ROCK2 are known to bind to GTPases RhoA and RhoC in their GTP-bound form, which in turn changes the conformation of ROCK and activates the kinase domain [25]. A small molecule screen performed by Moskal et al. showed a stark recruitment of parkin to damaged mitochondria in the presence of ROCK inhibitors. In particular SR3677, which inhibits ROCK2, promotes the beneficial events required for mitophagy including reduction of mitochondrial mass and lysosomal targeting of damaged mitochondria. They also found increased activity and mitochondrial presence of HK2 (hexokinase 2), a glycolytic enzyme previously involved in recruitment of parkin to damaged mitochondria [26].

Lastly, structural data on parkin brought about new insight into control of parkin activation and mitochondrial recruitment. It has been previously reported that binding of pUb (ubiquitin phosphorylated at S65 by PINK1) to phosphorylated parkin releases the catalytic activity of RING2 domain of autoinhibited parkin [27], 28], contributing to a feedback loop that enhances PINK1 activity and parkin recruitment to mitochondria. Parkin is also regulated by a feedforward mechanism. Sauvé et al. found that pUb binds to the RING1 domain with high affinity and to really interesting new gene 0 (RING0) domain with low affinity, which controls parkin’s localization and activation, respectively [29]. Interestingly, this mechanism can be activated independent of parkin’s phosphorylation at S65, and moreover, replacing the Ubl (ubiquitin-like) domain of parkin with ubiquitin is also suitable for PINK1 phosphorylation, underscoring the robustness of the PINK1/parkin pathway of mitophagy.

## Deubiquitinases (DUBs) in the regulation of parkin activity

Ubiquitination by the E3 ubiquitin ligase parkin is a key step in parkin-mediated mitophagy. DUBs remove or trim ubiquitin chains thereby counteracting ubiquitin ligase activity [30]. Naturally, DUBs that act together with parkin are highly appealing to research due to their therapeutic potential. Niu and colleagues found that the DUB USP33, which localizes to the outer mitochondrial membrane, targets parkin and removes specific ubiquitin conjugates off of parkin [31]. Here, USP33 negatively regulates parkin activity as it hampers its ability to mediate K63- and K48-linked ubiquitin chains.

The DUB USP14 has also been reported to regulate autophagy and to suppress proteasomal activity [32], 33]. Recent investigations revealed that inhibition of USP14 promotes mitophagy in iNeurons [34]. Despite the finding that this happens in a PINK1/parkin-independent fashion and relies on the E3 ubiquitin ligase MITOL, inhibition of USP14 in human parkin knockout neurons has a beneficial effect as it relieves mitochondrial defects.

USP30, a DUB anchored to mitochondria, has been earlier described to counteract parkin-mediated ubiquitination of mitochondria [35]. In this context, the authors identified mitochondrial substrates that were ubiquitinated and de-ubiquitinated by parkin and USP30, respectively. A recent study used USP30 in a skillful approach to manipulate mitophagy. A USP30 peptide, derived from its transmembrane region, was used to inhibit USP30’s activity [36]. By binding directly to USP30, the peptide leads to USP30 autoinhibition and stimulates mitophagy. Moreover, the blocking peptide contains an LC3 interacting motif (LIR), which mediates the binding to LC3, and ultimately accelerates the autophagosome formation. Another study took a similar approach and developed a USP30-specific compound inhibitor (FT3967385). Using USP30-deficient SHSY5Y cells and SHSY5Y cells treated with the USP30 inhibitor, the researchers identified USP30-sensitive ubiquitination events on depolarized mitochondria [37]. The ubiquitome revealed that several TOM (translocase of the outer mitochondrial membrane) proteins, voltage-dependent anion channel proteins and synaptojanin 2-binding protein are ubiquitinated. Also, inhibition of USP30 or USP30 loss leads to rapid accumulation of pUb, validating USP30 as an inhibitor of PINK1/parkin mitophagy. In an ntc Drosophila model (ntc=nutcracker, functional homologue of mammalian FBXO7), the search for conserved mechanisms in mitophagy, revealed yet another functional detail on USP30 [38] as ntc (FBXO7) promotes the ubiquitination of mitochondrial proteins opposing the action of USP30, thereby setting the stage for PINK1-stimulated mitophagy. Furthermore, while ntc mutants show similar phenotypes as compared to PINK1 and parkin mutants that include defects in climbing and flight activity, ntc overexpression counteracts parkin phenotypes. The results support the idea that ntc and FBXO7 are, to a certain extent, functional homologues and allow for the exploration of conserved mitophagic events in flies.

## Novel insight into PINK1 functioning

**Figure 2:**
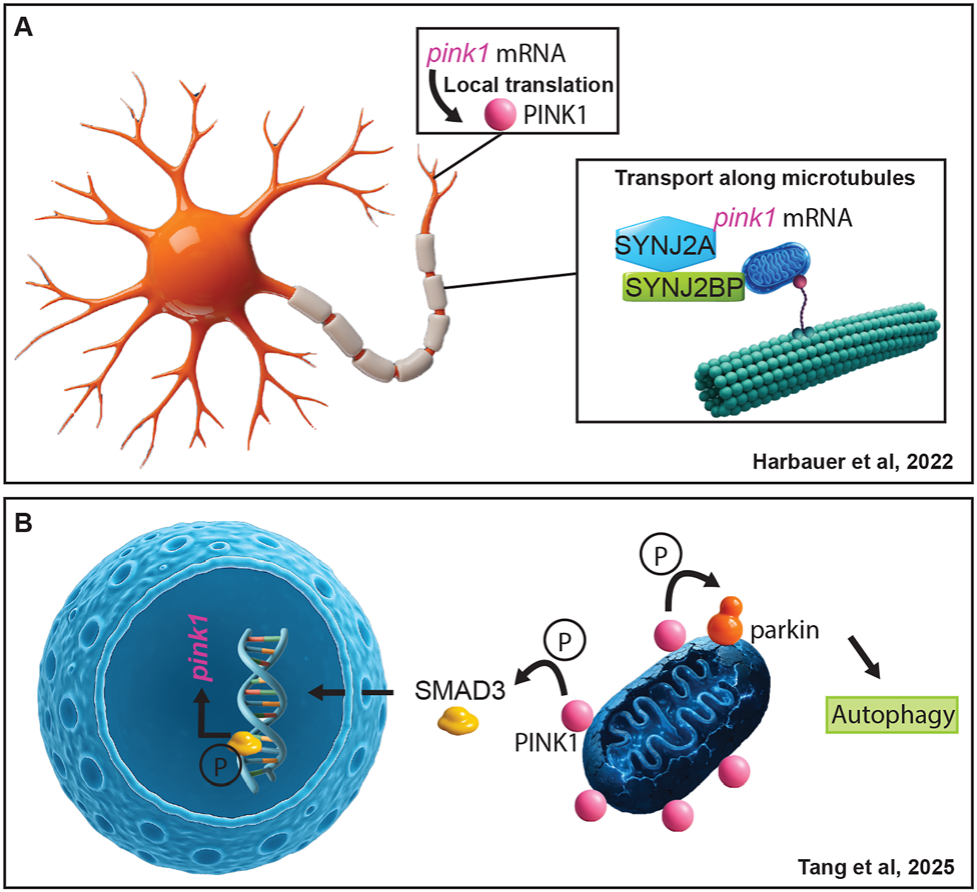
A. Harbauer and colleagues discovered a mechanism in which *pink1* mRNA travels together with mitochondria along microtubules for local translation. B. Tang and colleagues showed that the transcriptional modulator SMAD3 is phosphorylated by PINK1 and regulates *PINK1* gene expression.

It is well-known that PINK1’s half-life on healthy mitochondria is very short owing to the cleavage by mitochondrial proteases such as MPP and PARL (mitochondrial processing peptidase; rhomboid-like serine protease) in a membrane potential-dependent manner [39], [40], [41]. PINK1 is then expelled from mitochondria and degraded by the N-end rule. Thus, a valid question to ask is how neurons with long axons deal with the local availability of PINK1 for mitophagy if axonal transport occurs in the range of days. The intriguing answer is that *pink1* mRNA is transported along mitochondria in a neuron-specific manner [42], (Figure 2A). With the help of outer mitochondrial membrane proteins synaptojanin 2 binding protein (SYNJ2BP) and synaptojanin 2a (SYNJ2A), *pink1* mRNA is attached to the organelle, co-transported and translated locally. PINK1 levels are also subject to regulation on mitochondria as mitochondrial damage drives a positive feedback loop. Tang et al. identified SMAD3 (mothers against decapentaplegic homologue 3), a transcriptional modulator of the TGFbeta signaling pathway, as a target of PINK1, [43], (Figure 2B). SMAD3 pS423/425 however acts independently of its typical partners SMAD2 and SMAD4, as it translocates into the nucleus for transcriptional control of *pink1* expression. SMAD3 hence provides a prosurvival stimulus to the cell upon mitochondrial damage and the resulting upregulation of PINK1 level provides the seed for successful clearance of dysfunctional mitochondria.

Furthermore, while PINK1 on healthy mitochondria is rapidly degraded, depolarization leads to stabilization of PINK1 but also, in an early step of mitophagy, to the recruitment of the autophagic protein AMBRA1 (autophagy and beclin 1 regulator 1) to mitochondria. There, AMBRA1 interacts with PINK1 and ATAD3A (ATPase family AAA domain containing 3A), [44]. The latter regulates mitochondrial dynamics at the inner and outer mitochondrial membranes and has previously been shown to suppress PINK1-dependent mitophagy in (hematopoetic) stem cells [45], 46]. Interestingly, AMBRA1 stabilizes PINK1 as AMBRA1-silenced cells display decreased PINK1 levels [44]. Furthermore, PINK1 degradation is exacerbated by the mitochondrial matrix protease LONP1, which reportedly degrades PINK1 in addition to the aforementioned PARL [44], 47]. Hence, AMBRA1 represents an early regulatory element in PINK1/parkin mitophagy.

Another regulator of a more upstream step in mitophagy is TOM7 (mitochondrial import receptor subunit TOM7 homologue), a subunit of the translocase of the outer mitochondrial membrane (TOM) complex. Previously, the Youle lab identified TOM7 as a crucial player in the stabilization of PINK1 on the OMM upon loss of membrane potential [48]. Sekine and colleagues followed up and found that loss of TOM7 leads to the import of PINK1 into mitochondria regardless of the membrane potential. They further identified a short amino acid stretch adjacent to the transmembrane domain that is essential for PINK1’s kinase activity and hence parkin recruitment [49]. Alanine (A) mutations pinpointed a critical cluster of three glutamic acid (E) residues located in said stretch. A PINK1 3EA (3 glutamic acid residues mutated to 3 alanine residues) mutant abolishes PINK1’s autophosphorylation activity and it rendered PINK1 resistant to PARL-mediated degradation. Interestingly, in TOM7-deficient conditions, the inner membrane metalloendopeptidase OMA1 takes over and cleaves PINK1 upon mitochondrial depolarization. Taken together, a critical amino acid stretch allows for the accumulation of PINK1 on mitochondria, with TOM7 preventing PINK1’s import and OMA1 effectively cleaving PINK1 when falsely imported.

As described above, PINK1 phosphorylates ubiquitin at S65. In this context, Wall et al. characterized the protein phosphatase with EF hand domain 2 (PPEF2), that dephosphorylates pUb and counteracts PINK1’s phosphorylation [50]. Interestingly, mass spectrometry analyses revealed that several pUb-modified proteins, that emerge upon mitochondrial damage, are shared targets of PINK1 and PPEF2. PPEF2 thus acts as an inhibitor of mitophagy.

A fundamental aspect of PD research is also the understanding of how mutations in PINK1 meddle with normal mitophagy as this might be important for personalized therapeutic approaches in the future. A recent study took upon the task to examine several variants in PINK1 and in parkin in a systematic manner with enzymatic assays [51]. The authors selected both, variants with a minor allelic frequency of >1 % and rare variants (<1 %) and determined the CADD score (Combined Annotation Dependent Depletion, University of Washington, [52]), which provides information on the deleteriousness of a single nucleotide variant or other genomic alterations. Among the common variants, parkin S167N, located in the RING0 domain, displays a high CADD score and shows a delayed response to mitochondrial damage. Most rare parkin variants have high CADD scores and their phenotypes include low mitochondrial translocation rate, reduced pUb signals and reduced mitochondrial turnover. For PINK1, most of the rare variants have a high CADD score and two of them show loss of kinase activity consistent with the localization of the amino acid exchange in the kinase domain. In another study by the same lab, examination of residue G411 of PINK1 revealed that the pathogenic serine substitution reduced kinase activity [53], 54]. Interestingly, substitution experiments examining G411A leads to enhanced phosphorylation of ubiquitin by PINK1, rendering G411 a promising drug target.

## Upstream regulators of PINK1/parkin-mediated autophagy

**Figure 3:**
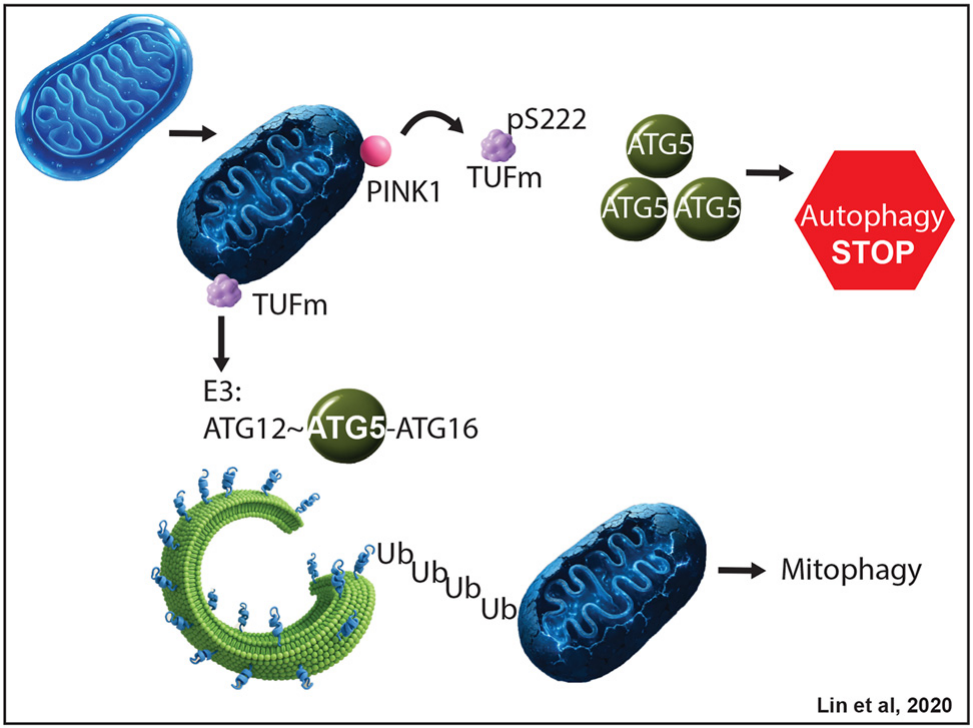
Lin and colleagues characterized TUFm as phosphoswitch in PINK1/parkin mitophagy. Upon phosphorylation, TUFm p222 inhibits mitophagy by sequestering ATG5. In contrast, unphosphorylated TUFm promotes mitophagy.

The regulation of PINK1 is of great complexity as recent work has identified novel players in the regulation of upstream events of autophagy. An interesting mechanistic feature of mitophagy involving PINK1 was discovered by Lin and colleagues, who characterized the interaction of PINK1 with the mitochondrial translation elongation factor Tu (TUFm), known as a GTP hydrolase active in protein biosynthesis, and additionally showed how TUFm controls E3 enzyme functioning [55]. Lin et al. found that PINK1 phosphorylates TUFm at S222 and that TUFm phosphoS222 quickly redistributes from mitochondria to the cytosol. In addition, they showed that while non-phosphorylatable TUFm promotes autophagic flux in cells, phosphomimetic TUFm blocks this effect, revealing TUFm as a phospho-switch in autophagy. Interestingly, the authors found that TUFm pS222 sequesters monomeric ATG5, a subunit of the E3 enzyme required for LC3 lipidation, a process facilitated by the LC3-conjugating system (ATG12∼ATG5-ATG16). Cytosolic TUFm pS222 thus exerts its action by inhibiting ATG12∼ATG5 E3 formation. Collectively, PINK1 and TUFm interact in the regulation of mitophagy independently of parkin, with TUFm acting as negative regulator (Figure 3).

The typical initiation step of autophagy requires the ULK1/2 initiation complex to activate the PI3K complex, comprising beclin1, VPS34, VPS15, ATG14, which generates phosphatidylinositol-3-phosphate (PI3P) for the nucleation of the isolation membrane. Selective autophagy receptors such as such as p62/sequestosome-1, NDP52 or neurally-expressed optineurin recognize ubiquitinated cargo destined for degradation, and they also recruit the ULK1/2 complex to engage the autophagy machinery.

Previous studies examined how receptors and cargo end up connecting with the isolation membrane. Tank-binding kinase 1 (TBK1, a serine/threonine kinase) has been identified as a crucial player in exactly this process as TBK1 phosphorylates several autophagic receptors including p62 and optineurin at autophagy-relevant residues located in their ubiquitin domains and LC3-interacting regions (LIR) [56], [57], [58]. Importantly, these phosphorylation events enhance the recruitment and tethering of damaged mitochondria to isolation membranes for selective autophagy. Further discoveries, shedding light on the upstream regulation of autophagy, were reported by Vargas and colleagues, who showed that the interaction of NDP52 and the ULK1 complex is mediated by FIP200, a coiled coil domain-harboring protein [59]. In this context, TBK1 stimulates the association of NDP52 and the ULK1 complex in a kinase activity-dependent manner and thus positively affects mitophagy. Also, NDP52 together with TBK1 are required in PINK1/parkin-mediated mitophagy. Recently, Nguyen et al. characterized an unconventional initiation scenario that is different from the machinery described by Vargas et al. Here, the experiments revealed an alternative initiation mechanism of mitophagy involving optineurin and TBK1 [60]. Remarkably, TBK1 acts in an ULK1 complex-independent manner to recruit the PI3K complex and ULK1/2 are dispensable for PINK1/parkin-regulated mitophagy, while the ULK1/2 complex subunits FIP200 and ATG13 are not. Taken together optineurin and NDP52 use different routes to initiate autophagy, showcasing the cell’s plasticity in mitophagy.

Strikingly, TBK1 is not only part of the initiation process but also active in close proximity to damaged mitochondria. Heo and colleagues previously described TBK1 as a dynamically activated kinase upon mitochondrial depolarization and RAB7A as a novel target of TBK1 during PINK1/parkin-dependent mitophagy [61]. Moreover, they found that phosphorylation of RAB7A at S72 mediates the binding to FNIP1 and its binding partner, the GTPase-activating protein FLCN, and that FLCN stimulates parkin-dependent mitophagy. A recent study by Tudorica and colleagues elaborated the molecular underpinnings of TBK1-mediated phosphorylation of RAB7A at S72 in mitophagy even further [62]. They found that phosphorylated RAB7A abrogates the interaction with rubicon, a RAB7A-binding proteins that negatively regulates autophagy. Phosphorylated RAB7A allows for the binding to pacer, a positive regulator of autophagy. Depolarized mitochondria are hence decorated with pacer-RAB7A pS72. Interestingly, knockout of pacer reduces parkin-mediated mitophagy, indicating the dependence of parkin on pacer-Rab7a pS72.

Another player at the isolation membrane is TOLLIP (Toll-interacting protein), or yeast homologue Cue5, a negative regulator of IL-1beta receptor signaling. TOLLIP (yCue5) has been identified as a highly conserved component in the recruitment of ubiquitinated cargo to LC3-decorated isolation membranes [63]. Here, TOLLIP (yCue5) serves as an adaptor between ubiquitin and LC3. Interestingly, Lu and colleagues also demonstrated that TOLLIP targets aggregation-prone proteins for autophagy implicating its role in neurodegeneration. In a recent study, TOLLIP was reported to associate with PINK1 and to enhance the binding of PINK1 to the mitochondrial protease MPPbeta, which stimulates the proteolytic processing of PINK1 [64]. TOLLIP also inhibits autophagy of damaged mitochondria, suggesting a potential blockade of PINK1-faciliated neuroprotection.

## *PARK* loci interacting with PINK1/parkin-regulated mitophagy

A logic step in PINK1/parkin-regulated mitophagy is to examine other proteins encoded by *PARK* genes. Interestingly, alpha-synuclein, encoded by *PARK1/4*, appears to have interactions with mitochondria as alpha-synuclein seeding events take place on mitochondria and emerging aggregates impair mitochondrial complex I [65], 66]. Recent studies also show that alpha-synuclein, whose elevated wild type protein levels alone can induce PD, is involved in mitophagy. Here, the study by Kinnart and colleagues showed that enhanced alpha-synuclein expression in several model systems suppresses mitophagic flux while non-mitochondrial autophagy remains unaffected [67]. Another study found that alpha-synuclein and PINK1/parkin appear to converge on mitophagy. With disease causing alpha-synuclein levels, both parkin levels and pUb levels are increased in cells [68]. A similar response of high pUb levels was also observed in alpha-synuclein mice by the researchers.

Mutations in leucine-rich repeat kinase 2 (LRRK2, *PARK8*) causes autosomal dominant, classical PD characterized by infrequent dementia and slow progression [69]. LRRK2 has been reported earlier to play a role in autophagy [70], 71]. A closer look at LRRK2 G2019S, a mutant which harbors increased kinase activity, shows enhanced mitochondrial aggregation and stagnating clearance of the organelles when met with a mitochondria-damaging protonophore [72]. These events are a consequence of failed interactions between TOM proteins and parkin, and the mitochondrial fission protein DRP1 (dynamin-related protein 1) and parkin in the early phase of mitophagy. A kinase-dead variant of LRRK2 or a specific LRRK2 inhibitor mitigates the aggregation of mitochondria. Interestingly, human fibroblast from patients carrying the LRRK2 G2019S mutation, display comparable defects. Another study found that human fibroblasts from patients with LRRK2 G2019S and R1441C mutations display impaired mitophagy [73]. While it does not affect the activity of PINK1 and parkin, the researchers found that it leads to a reduction in optineurin. Further analyses underscored the convergence of pathways by showing that LRRK2 G2019S and R1441C excessively phosphorylate the target RAB10 at S73. This results in failed interaction of RAB10 and optineurin, in failed accumulation of RAB10 and optineurin on depolarized mitochondria, and ultimately in failure to engage in mitophagy.

DJ-1 (*PARK7*), a parkinsonism-associated deglycase, has been associated with early-onset, autosomal recessive parkinsonism with slow disease progression [74]. It is involved in a plethora of functions including neuroinflammation, cell death, and interestingly in autophagy and mitochondrial dynamics [75]. A recent report examined the mechanistic underpinnings of mitochondrial turnover and found DJ-1 recruitment to damaged mitochondria in addition to impaired mitophagy in DJ-1-deficient cells [76]. The study also demonstrated that in the context of PINK1/parkin-regulated mitophagy, DJ1 acts downstream of their activation and recruitment process and facilitates the critical translocation of optineurin to mitochondria.

The D620N variant of vacuolar protein sorting-associated 35 (VPS35) protein has been previously implicated in autosomal dominant PD (*PARK17*), [77]. VPS35, together with VPS26 and VPS29, is a component of the retromer complex, which is a master regulator of endosome sorting [78]. Expression of VPS35 D520N in cells was found to affect the mitochondrial membrane potential at steady-state [79]. Furthermore, more than half of the mitochondria display swollen morphology and more severe defects reflected by a progressively deteriorating state of cristae. This leaves the cells unable to recruit PINK1/parkin to the damaged mitochondria demonstrating the convergence of several PD proteins on mitophagy.

Mutations in the F-box protein FBXO7 are causative for early-onset pyramidal-parkinsonian syndrome, a complex autosomal recessive variant of PD [80], 81]. A previous study presented the association of PINK1, FBXO7 and parkin and identified FBXO7 as a PINK1-interacting protein mediating the recruitment of parkin to mitochondria [82]. This model however was recently challenged by the Harper lab whose findings suggested that FBXO7 is dispensable for the recruitment process [83].

## Crosstalk of the ubiquitin proteasome system (UPS) and PINK1/parkin-regulated autophagy

The UPS is a complex enzymatic cascade responsible for the ubiquitination of cytoplasmic proteins. The reaction is initiated by the activation of the E1 ubiquitin-activating enzyme that mediates the transfer of ubiquitin to the E2 ubiquitin-conjugating enzyme in an ATP-depending manner, leading to the formation of the E2∼ubiquitin (E2∼Ub) conjugate. E2∼Ub and the substrate protein are recruited by the E3 ubiquitin ligase providing the physical proximity for the transfer of ubiquitin to the substrate. Ubiquitination comes in different variations and ranges from monoubiquitination, multi-monoubiquitination to polyubiquitination, which is viewed as a code by the cell. The responses include proteasome-mediated degradation for short-lived proteins or proteins whose disposal is mandatory such as cell cycle proteins. Moreover, ubiquitination results in functional modification, which is e.g. true for ubiquitin-mediated transmembrane protein internalization such as AMPA receptors [84], 85]. So, from a mechanistic point of view, the conjugation systems are shared concepts by the UPS and the autophagy process. Furthermore, the UPS and autophagic processes cooperate in cellular quality control. Here, the Arg/N-degron pathway has emerged as a novel mechanism, in which basically all amino acids could serve as N-terminal degrons of a protein, recognized by matching N-recognins, e.g. p62 or E3 ubiquitin ligases, resulting in the lysosomal or proteasomal degradation, respectively (see review [86]).

To investigate the connection of UPS and autophagy in the context of PINK1/parkin functioning, ionophore- and FCCP-induced removal of mitochondria was investigated in PINK1 knockout cell culture model by the Klein lab [87]. Both mitochondrial stressors lead to loss of membrane potential. While the removal of mitochondria is feasible even without LC3 lipidation upon valinomycin treatment, a working proteasome is required for mitochondrial clearance, suggesting a crucial dependence on the UPS during mitophagy. Further evidence revealed a role for the subunit PSMA7 in mitophagy and underscored the need of proteasomal proteins [88]. Here, PSMA7 interacts with the autophagy protein ATG5, required for LC3 conjugation, and with parkin while translocating to mitochondria. Both PSMA7 and the proteolytic subunit of the proteasome PSMB5 are required for the recruitment of ATG5 and parkin onto damaged mitochondria and for successful progression of PINK1/parkin-dependent mitophagy.

The role of the UPS in mitophagy was also investigated by Lechado-Terradas and colleagues who investigated steps that mitochondrial material goes through during parkin-dependent mitophagy. They identified the rapid degradation of some OMM proteins in a proteasome-dependent manner and the slower removal of ubiquitinated OMM proteins not targeted to proteasomal degradation but to autophagy [89]. In addition, they observed a delayed lysosomal degradation of IMM and matrix proteins but found that the proteasome affects degradation in the late stage of mitophagy as well, revealing complex parkin-dependent degradation dynamics during mitophagy.

Another intriguing interplay of the UPS and mitophagy was uncovered in the context of parkin-mediated apoptosis. A new line of evidence that implicates parkin in the decision of cell death, was e.g. reported by Ham et al. who showed that the ubiquitination status of the parkin substrate VDAC1, an OMM protein, tips the scales against mitophagy and in favor of apoptosis [90]. Quarato and colleagues picked up on parkin-mediated apoptosis and showed first of all that PINK1 is required for this phenomenon [91]. They furthermore demonstrated that BAK and BAX are not required for parkin-mediated cell death, while release of cytochrome c and activation of APAF1 occur. As an siRNA screen suggested a role for the proteasome in parkin-mediated cell death, the authors showed that inhibition of the proteasome or silencing of critical proteasomal subunits prevents apoptosis. Additionally, enrichment of autophagy-related pathways led to the finding that delayed autophagy also promoted parkin-mediated cell death.

## Analysis of PINK1/parkin-regulated mitophagy *in vivo* and treatment approaches

While much of the research has been established with *in vitro* experiments, it is highly important to verify the major findings *in vivo* and in doing so, respecting animal welfare. To validate impaired mitophagy *in vivo*, probes assessing mitochondrial health are useful, but may result in conflicting reports. A thorough study by Liu and colleagues directly compared mito-QC and mt-Keima, two popular fluorescent probes that differentiate between mitochondria and mito-lysosomes, both in cell culture and importantly in mice subjected to PINK1/parkin-activating mitochondrial stress [92]. The results demonstrated that mt-Keima is more sensitive in detecting mitochondrial stress in mice than mito-QC, revealing a superior performance of the former probe *in vivo*.

Another popular indicator of PINK1/parkin-mitophagy is the presence of pUb-decorated mitochondria, which are detectable with specific antibodies. While the detection in an artificial stress situation works well, the detection of physiological levels with previous antibodies is nearly impossible. So, the Springer lab developed highly specific, high-affinity pUb antibodies, which represent promising tools for *in vivo* analyses and diagnostics [93], 94]. In addition, the same lab addressed the understanding of basal levels of endogenous activation of PINK1 and parkin in rodent brains, in PD-derived cells and isogenic neurons [93], 94]. Analyses of parkin and pUb levels in *PINK1-/-*brain show an increase of the former and a decrease of the latter. *Parkin-/-*brains display also a decrease of pUb. Skin fibroblasts from PD patients with heterozygous or homozygous PINK1 Q456X (in C-lobe of the kinase domain) show little to no PINK1 under basal conditions and an upregulation upon mitochondrial stress in the heterozygous genotype only. Parkin levels show already an upward trend under basal conditions, mitochondrial stress however evokes a strong increase in parkin levels in the homozygous genotype only. A similar response of PINK1 is observed in WT and homozygous PINK1 I368N (in C-lobe of the kinase domain) skin fibroblasts when compared to PINK1 Q456X. PINK1 levels respond only in WT cells to mitochondrial stress but not in mutant cells. Parkin levels display lower levels in WT cells, without any discernible change in PINK1 I368N homozygous cells. Lastly, the comparison of I368N fibroblasts and I368N dopaminergic neurons, differentiated from the same genetic source, shows a stark increase of parkin levels in the latter. These results suggest that the response of parkin changes more drastically depending on the genetic constellation and cell type as compared to PINK1.

Since PINK1/parkin-regulated mitophagy poses a therapeutic target, the search for drugs to manipulate this pathway comes naturally. The small molecule BL-918 has been identified in a screen where it stood out as a strong stimulator of parkin translocation to mitochondria and was validated to efficiently promote mitophagy in cells [95], 96]. Importantly, administration of BL-918 in MPTP PD mice alleviates motor symptoms and histopathological phenotypes including tyrosine hydroxylase (TH) levels and number of TH-positive and dopamine transporter (DAT)-positive neurons. Interestingly, no such improvement is observable in *PINK1-/-* mice, underscoring the dependance on PINK1 by BL-918. Other newly developed compounds (BIO-200781 and BIO-197590) act as allosteric modulators of parkin that enhance its activity by gluing pUb to parkin [97]. Crystal structure analysis of the most active compound revealed that it tethers two pUb molecules to parkin. In addition, the authors showed that the compounds improve the activity of parkin mutants both in isolated organelles and cells. In cells, they reported a particular enhancement of parkin R42P and V56E, mutations which are causative of early-onset PD.

Ursodeoxycholic acid (UDCA), typically used to treat certain liver diseases, interferes with upstream events of the mitochondrial pathway thereby inhibiting apoptosis [98]. Qi and colleagues addressed whether or not UDCA shields neurons from damage and has beneficial effects in an MPTP PD mouse. They showed that in MPP^+^-treated cells, UDCA significantly lowers cell death rate, reverses the MPP^+^-induced response of BCL-2 proteins and positively affects mitochondrial health [99]. Furthermore, they demonstrated that MPTP PD mice perform better in motor tests and display more surviving dopaminergic neurons in the substantia nigra pars compacta. Another drug that was tested in the MPTP PD mouse model was Dynasore, which inhibits the GTPase activity of DRP1 and brings about fissogenic activity to mitochondria. Treatment of mice with Dynasore ameliorates their locomotor behavior, and while the treatment does not affect mitochondrial morphology, it increases the levels of PINK1 and parkin on mitochondria [100].

An interesting approach was reported by Wang and colleagues who examined a novel compound in an inflammasome PD mouse model [95], 96]. The inflammasome is a supramolecular complex consisting of the damage-sensing protein NLRP3, which belongs to the nucleotide-binding domain (NBD)- and leucine-rich repeat (LRR)-containing protein (NLR) family), (in-depth review: [101]). NLRP3 activates caspase-1, which triggers the activation of cytokines and thus inflammatory processes in the cell. Recent research implicated inflammasome action in PD (in-depth review [102]). Using wild type and *parkin+/−*mice, NLRP3 inflammasome activation was induced by LPS (lipopolysaccharide), a popular tool to trigger cellular inflammation [95], 96]. The mice were then treated with the specific NLRP3 inflammasome inhibitor MCC950. MCC950-treated wild type mice show enhanced activity of PINK1 and parkin, improved autophagy and inhibition of inflammation when compared to mice, that did not receive MCC950. Interestingly, while LPS treatment aggravates the pathologies in *parkin+/−*mice, MMC950 fails to improve the phenotype, indicating that the drug actually requires parkin and PINK1 to exert its effect.

Lastly, an intriguing study presented a potential acidification therapy in model systems, an approach that followed up on a previous finding that described that acidification of cytosolic pH triggers mitophagy [103]. Here, the researchers applied the ionophore nigericin, that acts as a potassium and proton antiport and that facilitates ion exchange across membranes. To take advantage of the acidification effect, a recent report by this lab used fibroblasts from healthy individuals and from PD patients harboring either a homozygous PINK1 mutation, a heterozygous PINK1/parkin mutation, or alpha-synuclein A53T or triplication [104]. These fibroblasts were treated with sodium lactate or sodium pyruvate as they reduce cytosolic pH. As a consequence of the acidified milieu, the cells display increased autophagic flux and lower cell death rates. Using brain slice cultures that were isolated from wild type and *PINK1-/-* mice and treated with lactate, the authors found an increase in mitophagy.

## Concluding remarks

In light of the dramatic increase of prevalence of PD and the burden on families and society that PD is associated with, it is most pressing to pursue PD research to enhance our understanding of the cellular processes contributing to or accelerating disease progression. Impaired mitophagy is without doubt a critical aspect of PD. The complexity is astounding as we continue to learn more about the many players involved in PINK1-parkin-regulated autophagy highlighted here, let alone further genes and mechanisms beyond the scope this minireview. It is crucial to know the modulators, in particular the intrinsic inhibitors of autophagy and how potential therapies interact with them. It is also important to understand how mutations and genetic variants affect autophagic pathways as development of therapeutic is expected to steer into a more personalized medicine. The curiosity and hard work in the research labs around the world are key to approach therapeutic goals, and give hope to those who are affected by PD.
